# Gamow Temperature in Tsallis and Kaniadakis Statistics

**DOI:** 10.3390/e24060797

**Published:** 2022-06-08

**Authors:** Hooman Moradpour, Mohsen Javaherian, Ebrahim Namvar, Amir Hadi Ziaie

**Affiliations:** Research Institute for Astronomy and Astrophysics of Maragha (RIAAM), University of Maragheh, Maragheh P.O. Box 55136-553, Iran; m_javaherian@znu.ac.ir (M.J.); ebrahim.namvar@gmail.com (E.N.); ah.ziaie@maragheh.ac.ir (A.H.Z.)

**Keywords:** generalized statistics, star formation, quantum tunneling, equipartition theorem

## Abstract

Relying on the quantum tunnelling concept and Maxwell–Boltzmann–Gibbs statistics, Gamow shows that the star-burning process happens at temperatures comparable to a critical value, called the Gamow temperature (T) and less than the prediction of the classical framework. In order to highlight the role of the equipartition theorem in the Gamow argument, a thermal length scale is defined, and then the effects of non-extensivity on the Gamow temperature have been investigated by focusing on the Tsallis and Kaniadakis statistics. The results attest that while the Gamow temperature decreases in the framework of Kaniadakis statistics, it can be bigger or smaller than T when Tsallis statistics are employed.

## 1. Introduction

In the framework of Maxwell–Boltzmann–Gibbs statistics, also known as Gibbs statistics, consider a gas with temperature $T_{g}$, including particles with mass *m* and mean velocity *v*. In this manner, using the equipartition theorem, one finds

(1) $$\begin{array}{c} \frac{1}{2}mv^{2}=\frac{3}{2}K_{B}T_{g}. \end{array}$$

Here, $K_{B}$ is the Boltzmann constant. For a pair of particles (called the *i*-th and *j*-th particles) with atomic numbers $Z_{i}$ and $Z_{j}$, respectively, located at a distance $r_{0}$ from each other, the Kinetic energy lets particles overcome the Coulomb barrier meaning that nuclear fusion begins, and consequently, a star is born if $\frac{3}{2}K_{B}T_{g}\geq U\left(r_{0}\right)=\frac{Z_{i}Z_{j}e^{2}}{4\pi \epsilon_{0}r_{0}}$ leading to [1]

(2) $$\begin{array}{c} T_{g}\geq \frac{Z_{i}Z_{j}e^{2}}{6\pi \epsilon_{0}K_{B}r_{0}}≃1\cdot 1\times 10^{10}\frac{Z_{i}Z_{j}}{r_{0}}\equiv T_{0}, \end{array}$$

for the gas temperature. On the other hand, for the temperature of the gas, we also have [1]

(3) $$\begin{array}{c} T\approx 4\times 10^{6}\left(\frac{M}{M_{⊙}}\right)\left(\frac{R_{⊙}}{R}\right), \end{array}$$

in which *M* ($M_{⊙}$) and *R* ($R_{⊙}$) denote the mass and radius of the gas (Sun), respectively. As an example, consider the Sun, for which we have $T\ll T_{0}$, meaning that the Sun should not burn [1]. Therefore, nuclear fusion cannot be launched in gasses whose temperature (T) are lower than $T_{0}$ (i.e., $T<T_{0}$) [1].

Thanks to the scorching Sun, the above argument becomes questionable. Indeed, Gamow is someone who was able to find a proper answer by proposing a mechanism: quantum tunneling [1]. Based on this theory, if the particles become close to each other as their de Broglie wavelength ($r_{0}≃\frac{\hbar }{p}\equiv \lambda$), then they overcome the Coulomb barrier. In this manner, the corresponding de Broglie wavelength of particles can be calculated as

(4) $$\begin{array}{c} \lambda =\frac{2\pi \epsilon_{0}\hbar^{2}}{mZ_{i}Z_{j}e^{2}}, \end{array}$$

where p=mv is considered, we replaced $r_{0}$ with $\lambda \equiv \frac{\hbar }{p}$, and then used

(5) $$\begin{array}{c} \frac{p^{2}}{2m}=\frac{Z_{i}Z_{j}e^{2}}{4\pi \epsilon_{0}\lambda }. \end{array}$$

Now, using $\frac{3}{2}K_{B}T_{g}\geq U\left(r_{0}\right)$, one reaches [1]

(6) $$\begin{array}{c} T_{g}\geq \frac{Z_{i}Z_{j}e^{2}}{6\pi \epsilon_{0}K_{B}\lambda }≃9\cdot 6\times 10^{6}Z_{i}^{2}Z_{j}^{2}\left(\frac{m}{\frac{1}{2}}\right)\equiv T, \end{array}$$

instead of Equation (2) meaning that nuclear fusion can be started in gases whose temperature are comparable with T (the Gamow temperature) not $T_{0}$ [1]. Moreover, using $\frac{3}{2}K_{B}T_{g}\geq U\left(r_{0}\right)$, one obtains

(7) $$\begin{array}{c} \lambda \geq \frac{Z_{i}Z_{j}e^{2}}{6\pi \epsilon_{0}K_{B}T_{g}}\equiv r_{0}^{T}. \end{array}$$

Now, bearing the equals sign in Equations (1) and (5) in mind, we can finally deduce that the minimum requirement for quantum tunnelling in a gas with temperature $T_{g}\geq T$ is $\lambda =r_{0}^{T}$. Hence, the equipartition theorem has a vital role, i.e., if it changes, then both Equation (6) and $r_{0}^{T}$ change. In summary, in one hand, it can be seen that T is comparable (and not equal) to T which justifies the burning of the Sun [1]. On the other hand, T decreases (increases) when $\frac{M}{R}$ is reduced (is enhanced) i.e., T varies from star to star [1]. Therefore, one may conclude that leaving the Gibbs statistics, a more flexible formula for T can be obtained which helps us to justify the burning of stars.

Although extensivity is the backbone of Gibbs statistics, there are various arguments in favor of the non-extensivity, especially in the relativistic systems and those that involve long-range interactions [2,3,4,5,6,7]. Tsallis and Kaniadakis (κ) statistics are two of the most famous and widely used generalized statistics frameworks [3,4,5,6,7] that propose generalized versions of the equipartition theorem [7,8,9]. Motivated by various reasons such as the long-range nature of gravity, and the probable relationship between the quantum aspects of gravity and the non-extensivity [9,10,11], these statistics have been employed to lead to notable outcomes in (i),describing dark energy [9,12], MOND theory [13], (ii) studying Jeans instability [14,15,16,17], and also (iii) stellar sciences [18,19,20,21,22].

Relying on the abovementioned achievements of Tsallis and Kaniadakis statistics, and the key role of the Gamow temperature in the stellar sciences, we are motivated to study the Gamow theory in these frameworks. Indeed, finding the Gamow temperature in Tsallis and Kaniadakis statistics is an important task that also helps one to obtain a better understanding of non-extensivity, gravity, and in fact, the non-extensive aspects of gravity. To achieve this goal, we focus on Tsallis and Kaniadakis statistics in the next section, and a summary will be presented at the end.

## 2. Generalized Statistics and the Gamow Temperature

### 2.1. Tsallis Framework

The Tsallis entropy content of a statistical distribution with *W* states while the *i*-th state happens with probability $P_{i}$ is defined as [5]

(8) $$\begin{array}{c} S_{q}^{T}=\frac{1}{1-q}\sum_{i=1}^{W}\left(P_{i}^{q}-P_{i}\right), \end{array}$$

where *q* is a free parameter calculated by other parts of physics or matching with experiments [4,5]. The Gibbs entropy is recovered at q→1; in fact, each sample has its own *q* [4,5]. For a three-dimensional particle, the ordinary thermal energy ($\frac{3}{2}K_{B}T$) is modified as $\frac{3}{5-3q}K_{B}T$, meaning that Equation (1) changes as

(9) $$\begin{array}{c} \frac{1}{2}mv^{2}=\frac{3}{5-3q}K_{B}T_{g}, \end{array}$$

where $0\leq q<\frac{5}{3}$ [9]. Now, simple calculations lead to

(10) $$\begin{array}{ccc} & & T_{g}\geq \frac{5-3q}{2}T\equiv T_{q}\Rightarrow 0<T_{q}\leq 2\cdot 5\;T,\\ & & \lambda_{q}=\frac{2\pi \epsilon_{0}\hbar^{2}}{mZ_{i}Z_{j}e^{2}}=\lambda , \end{array}$$

in which the subscript *q* is used to distinguish the previous results with those of the Tsallis statistics. Moreover, solving $\frac{3}{5-3q}K_{B}T_{g}=U\left(r_{0}\right)$, one reaches the Tsallis thermal length scale $r_{0q}^{T}\left(\equiv \frac{\left(5-3q\right)Z_{i}Z_{j}e^{2}}{12\pi \epsilon_{0}K_{B}T_{g}}=\frac{5-3q}{2}r_{0}^{T}\right)$ that meets the condition $r_{0q}^{T}\left(q\to 1\right)\to r_{0}^{T}$. Therefore, quantum tunneling can happen at a temperature comparable to $T_{q}$, and in this manner, we should have at least $r_{0q}^{T}=\lambda_{q}$.

### 2.2. The κ Statistics

In this framework, entropy is given by [6]

(11) $$\begin{array}{ccc} & & S_{\kappa }=-\sum_{i=1}^{W}\frac{P_{i}^{1+\kappa }-P_{i}^{1-\kappa }}{2\kappa }=\\ & & \frac{1}{2}\left(\frac{\sum_{i=1}^{W}\left(P_{i}^{1-\kappa }-P_{i}\right)}{\kappa }+\frac{\sum_{i=1}^{W}\left(P_{i}^{1+\kappa }-P_{i}\right)}{-\kappa }\right), \end{array}$$

leading to [12]

(12) $$\begin{array}{ccc} & & S_{\kappa }=\frac{S_{1+\kappa }^{T}+S_{1-\kappa }^{T}}{2}, \end{array}$$

which clearly testifies that the Gibbs entropy is achieved for κ=0 [3]. Indeed, κ is an unknown free parameter estimated by observations that varies from case to case [3]. Moreover, the equipartition theorem changes [7,9], and thus, Equation (1) takes the form

(13) $$\begin{array}{c} \frac{p^{2}}{2m}=\frac{3}{2}\gamma_{\kappa }K_{B}T_{g}, \end{array}$$

in which

(14) $$\begin{array}{c} \gamma_{\kappa }=\frac{\left(1+\frac{\kappa }{2}\right)\Gamma \left(\frac{1}{2\kappa }-\frac{3}{4}\right)\Gamma \left(\frac{1}{2\kappa }+\frac{1}{4}\right)}{2\kappa \left(1+\frac{3\kappa }{2}\right)\Gamma \left(\frac{1}{2\kappa }+\frac{3}{4}\right)\Gamma \left(\frac{1}{2\kappa }-\frac{1}{4}\right)}, \end{array}$$

where $0\leq \kappa <\frac{2}{3}$ and Γ(n) denotes the Gamma function [9]. Moreover, $\gamma_{\kappa }$ diverges for $\kappa =\frac{2}{3}$ and the ordinary equipartition theorem ($\frac{3}{2}K_{B}T$), and thus Equation (1) are recovered when κ=0 leading to $\gamma_{\kappa }=1$ [9].

Finally, it is a matter of calculation to find the Kaniadakis counterpart of Equation (10) and the Kaniadakis length scale as

(15) $$\begin{array}{ccc} & & T_{g}\geq \frac{T}{\gamma_{\kappa }}\equiv T_{\kappa },\\ & & \lambda_{\kappa }=\frac{2\pi \epsilon_{0}\hbar^{2}}{mZ_{i}Z_{j}e^{2}}=\lambda , \end{array}$$

and

(16) $$\begin{array}{c} r_{0\kappa }^{T}=\frac{r_{0}^{T}}{\gamma_{\kappa }}, \end{array}$$

respectively. Since $1\leq \gamma_{\kappa }$ [9], the conditions $T_{\kappa }\leq T$ and $r_{0\kappa }^{T}\leq r_{0}^{T}$ are obtained as the allowed intervals for $T_{\kappa }$ and $r_{0\kappa }^{T}$.

## 3. Conclusions

Reviewing the Gamow theory shows the role of equipartition theorem in more clarification via defining a thermal length scale ($r_{0}^{T}$). It was deduced that the nuclear fusion would occur in a gas whose temperature is comparable to the Gamow temperature (T) if the minimum requirement $\lambda =r_{0}^{T}$ is satisfied. Moreover, equipped with the fact that generalized statistics modifies the equipartition theorem and motivated by their considerable achievements in various setups [9,10,11,12,13,14,15,16,17,18,19,20,21,22], we studied the Gamow temperature within Tsallis and Kaniadakis statistics. The results indicate that the Gamow temperature (T) decreases in Kaniadakis statistics ($T_{\kappa }\leq T$), and in Tsallis statistics it can be smaller or bigger than T (i.e., $0<T_{q}\leq 2\cdot 5\;T$), depending on the value of *q*. The same result applies to the corresponding thermal length scales.

Correspondingly, it may be claimed that stars whose temperature T differs from T are signals of the non-extensive features of stellar sciences, meaning that if stars obey Tsallis (Kaniadakis) statistics, then by using $T=T_{q}$ ($T=T_{\kappa }$), one can find the value of *q* (κ) corresponding to each star. Hence, the upper and lower bounds on the *q* (κ) parameter for nuclear fusion process occurring can be found in the coldest and hottest stars. Finally, we should note that further theoretical studies and also fitting with observations are needed to determine the final probable generalized statistics governing the stellar sciences.

## Data Availability

This manuscript has no associated data or the data will not be deposited.
